# Polypoid Cystitis in an Adult without History of Catheterization

**DOI:** 10.5812/kmp.iranjradiol.17351065.3145

**Published:** 2011-11-25

**Authors:** Jae Eun Roh, Bum Sang Cho, Min Hee Jeon, Min Ho Kang, Seung Young Lee, Hyung Geun Song

**Affiliations:** 1 Department of Radiology, Cheongju Medical Center, Cheongju, Korea; 2 Department of Radiology, Chungbuk National University Hospital, Cheongju, Korea; 3 Department of Pathology, Chungbuk National University Hospital, Cheongju, Korea

**Keywords:** Tomography, X-Ray Computed, Urinary Bladder, Cystitis

## Abstract

Polypoid cystitis is a benign exophytic mucosal lesion of the bladder. Differentiating it from papillary transitional cell carcinoma is difficult due to their similar characteristics. Although indwelling catheter is the main well-known cause of polypoid cystitis, some case reports unrelated to catheterization have been described. However, the radiological findings of polypoid cystitis have rarely been reported. We hereby describe polypoid cystitis in a 20-year-old man without a history of catheterization along with the computed tomographic findings.

## 1. Introduction

Catheter-associated polypoid cystitis is a well-recognized condition that mimics bladder cancer [1][2]. However, polypoid cystitis in the absence of catheterization is extremely rare; thus, so far, less than 25 cases of polypoid cystitis unrelated to an indwelling catheter have been reported, with the majority of these reports describing pathological or cystoscopic findings [1][3][4][5]. We here describe our experience of a case of polypoid cystitis mimicking well-differentiated transitional cell carcinoma in a patient without a history of catheterization with the associated computed tomographic findings and a literature review.

## 2. Case Presentation

**Figure 1:**
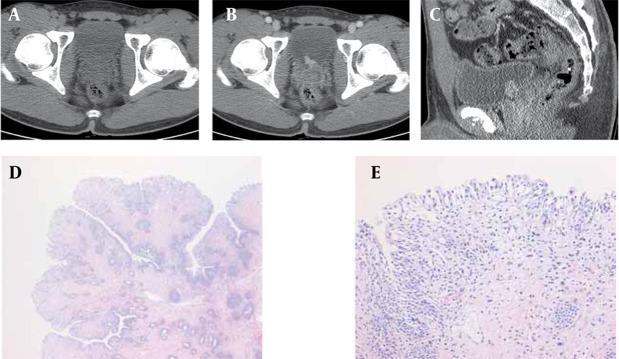
Computed Tomography Findings in a 20-Year-Old Man With Polypoid Cystitis. A, Pre-contrast axial scan shows no visible mass in the urinary bladder. B, Axial and C, Sagittal reformatted image show a polypoid homogeneously enhancing mass on the posterior wall of the urinary bladder near the bladder neck. D, Photomicrograph, broad-based polypoid architecture of the mass is noted. The stroma is mildly edematous (H and E stain, × 40). E, In the photomicrograph, the mass is lined by normal urothelium. Mildly edematous changes and infiltration of lymphocytes and plasma cells are observed in the stroma. Dilatation of the capillaries is also observed (H and E stain, × 200).

A 20-year-old man complaining of dysuria and straining on voiding for 6 months and gross hematuria for one month was admitted to our hospital. Serum urea, creatinine, electrolyte, white blood cells and hemoglobin values were normal. Urinalysis showed 20-29 RBC/HPF and 20-29 WBC/HPF. Urine culture and urine cytology were negative. Pre-contrast Computed tomoyraphy (CT) revealed no mass in the bladder. However, post-contrast CT demonstrated an intraluminal protruding polypoid lesion 1.5 cm in diameter on the posterior wall of the urinary bladder near the bladder neck. The lesion was homogeneously enhanced and no evidence of muscle layer invasion was observed (Figure 1 A-C). The radiological diagnosis was well-differentiated papillary transitional cell carcinoma and cystoscopy was then performed. Cystoscopy confirmed the papillary lesion on the right side of the bladder neck and transurethral resection of the mass was performed. Microscopic analysis revealed the presence of broad-based polypoid surface epithelium, edematous stroma, ectatic blood vessels and some inflammatory cells. No malignant cells were observed. The final diagnosis was polypoid cystitis (Figure 1 D and E). After operation, medical treatment was performed and the symptoms improved.

## 3. Discussion

Polypoid cystitis is a form of chronic cystitis characterized by an exophytic inflammatory lesion of the bladder mucosa [1]. Friedman and Ash [6] defined the diagnosis of “polypoid cystitis” in 1959 for the first time. The most well-known etiology of this condition is long-standing, indwelling catheterization [1][4][5][7]. This pathology occurs equally in men and women ranging in age from 20 months up to 79 years [7]. Ekelund et al. [2] reported the presence of its characteristic histological changes in 40 of 51 patients treated with urethral catheterization. They also reported that the frequency of polypoid cystitis increased with time, reaching its maximum at 3 months of catheterization [2]. However, theoretically, any factor that irritates the bladder mucosa may result in polypoid cystitis. Polypoid cystitis has also been noted to be associated with colovesical fistulas, calculi, urinary tract obstruction and a history of radiation therapy [4]. In our patient, there was no history of such other causes, including catheterization, fistula, calculi, urinary tract obstruction or radiation therapy. The radiological findings of polypoid cystitis are not well known. CT findings in polypoid cystitis are known to be nonspecific and cannot be differentiated from those of transitional cell carcinoma [1]. To our knowledge, only two reports have described the radiological findings of polypoid cystitis [1][3]. Kim et al. [1] and Choi et al. [3] reported the magnetic resonunce imaging (MRI) and CT findings of polypoid cystitis unrelated to indwelling catheterization. The size of the masses reported were approximately 5 cm and 15 cm and the masses were located at the lateral wall and the posterior wall of the urinary bladder, respectively. However, in our patient, polypoid cystitis manifested as a small polypoid lesion located at the posterior wall around the bladder neck. This finding was similar to those described by previous pathological and urological findings wherein the size of polypoid cystitis ranged from 0.7 cm to 3 cm [5][8][9]. These reports noted that the location of the lesion may aid in the differential diagnosis. Polypoid cystitis has mostly been observed on the dome and the posterior wall of the urinary bladder, an area in close contact with the catheter tip; moreover, bladder tumors are known to be rare in these locations [2][5]. It is believed that mechanical irritation by the catheter tip and the material of the catheter cause the development of polypoid cystitis [1][2]. In previous cases reported to be unrelated to indwelling catheters, the location of the mass has varied. Killic et al. [5] reported the clinical and pathological features of eight patients with polypoid cystitis unrelated to indwelling catheterization, noting that the lesions may be localized at different sites of the bladder and not only on the posterior wall and dome. Kim et al. [1] also reported a laterally located mass. We consider that the location of the mass in our patient was also an incidental finding; moreover, we believe that polypoid cystitis may present at any location in the urinary bladder similar to that described previously by Kim et al. [1] and Killic et al. [5].

The enhancement pattern of polypoid cystitis is not well known. In our patient, the mass was homogeneously enhanced. Kim et al. [1] did not mention the CT enhancement patterns in their report; however, on MRI, it appeared as a low-signal intensity mass on T2-weighted imaging and iso-signal intensity mass as compared with the bladder wall on T1-weighted imaging. Relatively high signal intensity with a branching pattern on T2-weighted imaging and mild enhancement was noted at the central portion of the mass, which correlated to the edematous fibrous core. MRI was not performed for our patient.

As described by Kim et al. [1] and Choi et al. [3], an exophytic growth pattern was observed in the histopathological examination of the resected mass in our case. This finding corresponds to the pathological definition of polypoid cystitis. Polypoid cystitis is a reversible, exophytic, inflammatory lesion of the bladder mucosa that is histologically characterized by normal or mildly hyperplastic urothelium, overlying a congested, chronically inflamed and markedly edematous stroma, but with metaplasia being rarely found [1][5]. Polypoid, papillary and bullous cystitis are considered to be a part of a continuous spectrum with identical pathological findings [1][5][8][10] , depending on the amount of stromal edema and gross morphologic characteristics of the exophytic lesions [1][5][8]. The papillary type has the narrowest base and may form an exophytic mass. The width of a bullous lesion is more than its height showing a gradual transition from the normal mucosa, which may differentiate this entity from bladder cancer [1][8]. It may be difficult to distinguish papillary-polypoid cystitis from various papillary urothelial neoplasms on radiological or microscopic examination and also at cystoscopy due to the similar appearances of the lesions, especially in patients with no history of catheterization [4][5][8][10]. However, polypoid cystitis generally manifests as small and multiple masses [1]. In our case, a solitary lesion was noted, necessitating transurethral resection of the mass after being misdiagnosed as transitional cell carcinoma on CT and cystoscopy. In the macroscopic and microscopic view, polypoid cystitis has much wider fronds in comparison with papillary carcinoma. Differentiating thin papillae of papillary cystitis from carcinoma is even more intricate [10]. As a result, it is necessary to resect all polypoid and papillary lesions in patients with or without a history of catheterization for microscopic examination to reach the definite differential diagnosis [5].

In summary, when a polypoid lesion in the urinary bladder is observed in a patient without a history of catheterization, polypoid cystitis must be considered as a differential diagnosis, especially in stalked lesions without evidence of bladder wall invasion.
